# Trajectories of loneliness and objective social isolation and associations between persistent loneliness and self-reported personal recovery in a cohort of secondary mental health service users in the UK

**DOI:** 10.1186/s12888-021-03430-9

**Published:** 2021-08-23

**Authors:** Ruimin Ma, Jingyi Wang, Brynmor Lloyd-Evans, Louise Marston, Sonia Johnson

**Affiliations:** 1 Division of Psychiatry, University College London, 6th Floor, Maple House, 149 Tottenham Court Road, London, W1T 7NF England; 2 Department of Psychological Medicine, Institute of Psychiatry, Psychology and Neuroscience (IoPPN), King’s College London, London, UK SE5 8AB; 3 Key Laboratory of Public Health Safety, NHC Key Laboratory of Health Technology Assessment, School of Public Health, Fudan University, Shanghai, 200032 China; 4 Department of Primary Care and Population Health, University College London, Rowland Hill Street, London, NW3 2PF UK; 5 Camden and Islington NHS Foundation Trust, St Pancras Hospital, 4 St Pancras Way, London, NW1 0PE England

**Keywords:** Loneliness, Social isolation, Mental health, Personal recovery, Psychiatry

## Abstract

**Background:**

Loneliness is a frequent and distressing experience among people with mental health problems. However, few longitudinal studies have so far investigated the trajectories of loneliness and objective social isolation, and the extent to which both issues may impact mental health outcomes among mental health service users. Therefore, this study aims to describe the trajectories of loneliness and objective social isolation and their associations with self-rated personal recovery among people leaving crisis resolution teams (CRTs).

**Methods:**

A total of 224 participants receiving care from CRTs (recruited for a large multi-site randomised controlled trial) were included in this longitudinal cohort study. They completed the eight-item University of California at Los Angeles Loneliness Scale (ULS-8), Lubben-Social Network Scale (LNSN-6), and the Questionnaire about the Process of Recovery (QPR) (primary outcome) at baseline, 4- and 18-month follow-up, as well as baseline sociodemographic and clinical variables.

**Results:**

We compared groups who were persistently lonely (at all time points), intermittently lonely (at one or two time points) and never lonely. After adjusting for all potential confounders and baseline predictive variables, persistent severe loneliness was associated with worse personal recovery at 18-month follow-up compared with the never lonely (reference group) (coef. = − 12.8, 95% CI -11.8, − 3.8, *p* < .001), as was being intermittently lonely (coef. = − 7.8, 95% CI -18.8, − 6.8, p < .001). The persistently objectively social isolated group (coef. = − 9.8, 95% CI -15.7, − 3.8, *p* = .001) also had poorer self-rated recovery at 18-month follow-up than those who were not socially isolated at any timepoint (i.e., reference category).

**Conclusion:**

Results suggest that both persistent loneliness and objective social isolation are associated with poorer self-rated recovery following a crisis, compatible with a causal relationship. These findings suggest a potential role for interventions aimed at alleviating loneliness and objective social isolation in improving recovery outcomes for people with mental health symptoms. Increased awareness of both issues among health practitioners is also warranted.

**Supplementary Information:**

The online version contains supplementary material available at 10.1186/s12888-021-03430-9.

## Introduction

Social relationships are fundamental to individuals’ emotional, behavioural and cognitive development [1]. Peplau and Perlman [2] define loneliness as a distressing experience that results from a perceived discrepancy between an individual’s desired and actual social relationships. Loneliness is thus a subjective experience [3]. Objective social isolation describes the quantitative aspect of individuals’ social relationships, such as the number of social relationships or the frequency of social contacts [4]. Wang and colleagues’ [5] conceptual review put forward a comprehensive framework on loneliness and its related constructs in relation to mental health, including this distinction between loneliness and objective social isolation.

Multiple studies have shown a significant impact of loneliness and objective social isolation on our mental health, including depressive symptoms [6], onset of psychotic symptoms [7], and decline in cognitive functioning [8]. Loneliness is a more pervasive problem among people with most mental health conditions than among the general population [9]. Objective social isolation (e.g., having fewer social contacts) is also commonly experienced by people with mental disorders [10]. The underlying causes of loneliness and objective social isolation in people with mental health problems are likely to be multifactorial, including social anxiety or difficulties in initiating and maintaining social relationships with others [11, 12], and discrimination and interpersonal stigma toward people with mental health symptoms [13, 14].

Supporting personal recovery is increasingly acknowledged internationally as a key goal of mental health care and mental health policy [15, 16]. In mental health, the term ‘recovery’ now tends to be multidimensional; it focuses on mental health service users’ personal experiences and personal goals for recovery [17]. The relational dimension of ‘recovery’ underscores the importance of maintaining interpersonal relationships and social contacts with family and friends [18]. While recent studies suggest a significant association between loneliness, poor social support and personal recovery among mental health service users [19], there is not as yet much evidence exploring longitudinal aspects of such associations and the likely direction of causality. Loneliness often is a transient experience; however, recent research has given grounds for particular concern about a sub-group of people who experience persistent loneliness, potentially related to factors such as maladaptive cognitive biases, social vigilance and specific attribution styles come into play [20]. Taking into consideration the association between loneliness, objective social isolation and a wide range of mental health outcomes, we should expect that being lonely or objectively socially isolated for an extended period of time may contribute to worse clinical outcomes in mental health service users. Yet, no research to date has investigated the importance of persistent loneliness or objective social isolation in mental health outcomes across a range of mental health diagnoses.

Given this knowledge gap, the aim of the current study was to advance our understanding of the persistence of loneliness and objective social isolation in a sample of people with mental health diagnoses, and whether people with persistent loneliness or objective social isolation have poorer personal recovery following recovery from a crisis. We studied a sample being discharged from crisis resolution teams (CRTs), which provide intensive home treatment as an alternative to hospital admission [21, 22]. We used data collected as part of a large randomised controlled trial (RCT) [23]: measures of loneliness and social isolation were not a main focus in reporting of this trial. Two previous papers have focused on loneliness and published analyses using baseline and 4-month follow-up data [24, 25], however, the current paper is the first to report on 18-month follow-up outcomes, and on those who are severely lonely or objectively socially isolated at multiple timepoints. By utilising the 18-month follow-up data, the current study could not only explore the trajectory of loneliness and objective social isolation over an 18-month period, but also examine the longitudinal association between loneliness, objective social isolation and self-rated personal recovery. Therefore, the current study had two main aims: 1) to identify between-group differences in sociodemographic and psychiatric characteristics between groups defined by whether they were persistently (i.e. being significantly lonely across three follow-up timepoints), intermittently (i.e. being significantly lonely at one or two follow-up timepoints) or never lonely (i.e. never being lonely across three follow-up timepoints), and between groups defined by whether they were persistently (i.e. being socially isolated across three timepoints), intermittently (i.e. being socially isolated at one or two timepoints) or never (i.e. never being socially isolated across three timepoints) socially isolated and 2) to examine whether persistent loneliness and persistent social isolation were associated with poorer self-rated personal recovery over an 18-month period. We hypothesised that 1) participants with persistent severe loneliness would have the poorest self-rated personal recovery at 18-month follow-up, followed by participants who suffered from intermittent severe loneliness, and then participants who were never severely lonely; and 2) Participants with persistent objective social isolation would have the poorest self-rated personal recovery at 18-month follow-up, followed by participants who suffered from intermittent objective social isolation, and then participants who were never objectively socially isolated.

## Method

### Participants

Participants were drawn from the Crisis Team Optimisation and Relapse Prevention (CORE) study. This large multi-site randomised controlled trial was primarily designed to test the effectiveness of a peer-provided self-management intervention versus treatment as usual supplemented by a self-management booklet on readmission rate for people with mental health problems, delivered following discharge from CRTs in six NHS trusts, including inner city, suburban and rural environments. Trial inclusion criteria and recruitment procedures are fully described in the published trial protocol [23]. For this paper, we pool participants in both arms of the trial to form a single cohort. A detailed baseline description of this cohort and the variations associated with loneliness at baseline has been previously published, however, in the original RCT, there was no evidence for an effect of the intervention on the primary outcome of the current study (i.e., self-rated personal recovery) [26].

Inclusion criteria for the CORE study required participants to have the capacity to provide written informed consent to participate and to have received support from one of the CRTs for a week or longer. Exclusion criteria included: 1) being clinically assessed as being too high risk to others to participate safely in the study or trial intervention; 2) lacking sufficient knowledge in English to complete the assessments; and 3) CRT discharge having occurred more than a month ago.

The CORE study was approved by the London Camden and Islington Research Ethics Committee (ref 12/LO/0988) before the commencement of data collection. Written informed consent was received from all participants prior participation. All methods were performed in accordance with the relevant guidelines and regulations.

### Measures

All participants recruited for the CORE study (i.e., both experimental and control groups) were assessed at three timepoints using structured interviews: baseline, 4- and 18-month follow-up. Sociodemographic information was only collected at baseline. The measures used in this research are reported below.

#### Social isolation variables

##### Loneliness

The University of California at Los Angeles (UCLA) Loneliness Scale (ULS-8) was administered as the main measure of loneliness. The ULS-8 consists of eight self-reported items, all rated on a 4-point Likert scale. The total score of this scale ranges from 8 and 32, and a higher score indicates more severe loneliness. This scale has demonstrated good validity and reliability (Cronbach’s alpha α = 0.84) [27]. For the current research, a participant was considered as being significantly lonely if his/her total score on the ULS-8 was 24 or above. This cut-off point was defined as scoring an average of three on each item, which is equivalent to being lonely at least sometimes. Trajectories of loneliness were determined based on loneliness scores at baseline, 4- and 18-month follow-up: 1) persistently severely lonely, included participants who scored as severely lonely at all three timepoints; 2) intermittently severely lonely, consisting of participants who scored as severely lonely at one or two timepoints only; and 3) never severely lonely, including participants who did not score as severely lonely at any timepoint.

##### Objective social isolation

The Lubben Social Network Scale (LSNS-6) is the revised version of the original LSNS. The LSNS-6 was administered to measure objective social isolation, and it aims to examine the number of kin relationships and non-kin relationships that participants keep in contact with, as well as the perceived quality of these relationships. The LSNS-6 has shown excellent convergent validity and internal consistency (Cronbach’s alpha α =0.83) [28]. LSNS-6 included six questions in total, with three questions evaluating individuals’ kin relationships and another three comparable questions evaluating their non-kin relationships. Out of these six questions, only item 1 and 4 aim to measure objective social isolation (i.e., the number of relatives and of friends one sees or hears from at least once a month) and the other items measure the perceived quality of social support received from these resources. Therefore, for the current study, only the two items relating to social network size were utilised as a measure of objective social isolation, which is conceptually distinct from loneliness. The sum of item 1 and 4 ranged from 0 to 10, with a higher score indicating lower objective social isolation.

#### Outcome measure (primary outcome)

##### The questionnaire about the process of recovery (QPR)

The QPR contains 22 items and yields a total score between 0 and 88. The higher the score, the better the self-rated intrapersonal and interpersonal recovery. The QPR has well-established psychometric properties [29], including great stability across time, satisfactory internal consistency, construct validity and test-retest reliability [30] (intrapersonal subscale r = 0.874, interpersonal subscale r = 0.769) [31].

#### Sociodemographic and psychiatric (controlled) variables

Participants’ sociodemographic and clinical characteristics were recorded at baseline, including age, gender, ethnicity, marital status, employment status, educational attainment, whether they were in contact with children under 16 years old, current diagnoses, number of psychiatric inpatient hospitalisation, and number of years since first contact with mental health services. Psychiatric symptoms were measured by the Brief Psychiatric Rating Scale (BPRS), which was developed to evaluate major symptoms for psychiatric patients [32].

### Statistical analyses

Case mean substitution was implemented to replace missing data on continuous variables, including loneliness, social network size and personal recovery. Given the low level of missing data on all scales at both baseline and 18-month follow-up in the current study, case mean substitution should have equivalent effectiveness as other missing data techniques [33]. Following established guidance [34, 35], cases with over 25% of data missing on a single scale were excluded from the final analysis. Pearson’s correlation was used to check for potential collinearity between all baseline independent variables.

T-test and chi-square tests were used to check predictors of missingness for the outcomes. When baseline variables were found to be different between the participants who completed the ULS-8 and LSNS-6 at all three timepoints (i.e., completers) and those who did not (i.e., non-completers), these variables were controlled for in the final analysis. The primary outcome, that is the association between the three loneliness groups and personal recovery at 18-month follow-up was tested by multivariable linear regression analyses. Firstly, a univariate linear regression model was carried out. Then four blocks of baseline variables were added into the multivariable regression models, in the following steps: 1) baseline social network size; 2) baseline sociodemographic variables (i.e. age, gender, ethnicity, educational attainment, and whether employed or in education or not) and baseline social network size; 3) baseline sociodemographic variables, baseline psychiatric variables (i.e. number of years since first contact with mental health services, the Brief Psychiatric Rating Scale (BPRS) total score, number of psychiatric inpatient hospitalisation), and baseline social network size; and 4) baseline sociodemographic and clinical variables, baseline social network size, and baseline QPR score. Comparable five steps were also carried out to examine the association between the three objective social isolation groups and personal recovery at 18-month follow-up.

All analyses were performed in STATA 12.1. Tests for two-sided significance at the *p* = 0.05 level were used.

## Results

### Baseline descriptive results

In total, 399 participants were included in the baseline analysis. Baseline characteristics of our sample are described in Additional file 1. A detailed description of participants at baseline has been previously published [24]. Of these 399 participants, only 224 participants completed measures at all three timepoints and could be included in the final analysis for research question 2. Please see Table 1 for the characteristics of participants at 18-month follow-up. The mean age of these participants was 40.0 (SD = 12.5), and about 40% were male. Approximately 30% of this sample was diagnosed with schizophrenia or other psychotic disorders. The mean of the QPR reported by these participants was 43.3 (SD = 10.7). Reported loneliness at 18-month follow-up had a mean score of 22.1 (SD = 4.5), and the mean of 18-month social network size was 5.0 (SD = 2.3).

**Table 1:** Characteristics of participants at 18-month follow-up

Variables	Participants at 18-month follow-up (***n*** = 224)	
	Mean (SD) or %	N
**Age**	39.97 (12.54)	224
**Gender (%)**		
Male	39.73%	89
Female	60.27%	135
**Ethnicity**		
White British/Irish/other	61.88%	138
Black, Black British/Caribbean/African/other	21.52%	48
Asian, Asian British/Indian/Pakistani/Bangladeshi/ other, Chinese	7.62%	17
Mixed White/Black Caribbean, Mixed White/Black African, mixed White/Asian, other mixed, other ethnic groups	8.97%	20
**Marital status**		
Single/Separated/divorced/widowed	74.55%	167
Married/cohabiting	25.45%	57
**UK born**		
No	24.89%	55
Yes	75.11%	166
**Housing**		
Permanent/supported accommodation	96.88%	217
Unstable accommodation	3.13%	7
**Contact with children under 16**		
No contact	6.25%	14
Contact with dependent children	22.32%	50
Having no children	71.43%	160
**Educational attainment**		
No qualification	15.25%	34
Other qualification	50.67%	113
Degree	34.08%	76
**Employment/education status**		
Not in employment/education/full time caring role	43.89%	97
Yes	56.11%	124
**Loneliness score**	22.07 (4.50)	224
**Social network size**	4.97 (2.28)	224
**Number of psychiatric inpatient hospitalisations**		
Never	62.50%	140
Once	15.63%	35
2 or more	21.88%	49
**Number of years since first contact mental health services**		
Less than 3 months	16.07%	36
3 months – 2 years	16.07%	36
2–10 years	33.93%	76
More than 10 years	33.93%	76
**Current diagnosis**		
Schizophrenia or schizoaffective disorder/bipolar affective disorder/other psychosis	30.14%	66
Depression/anxiety disorder/post-traumatic stress disorder	26.94%	59
Borderline or emotionally unstable personality disorder/other personality disorder	11.42%	25
Other diagnosis	31.51%	69
**BPRS total score**	43.30 (10.67)	223
**QPR total score**	51.21 (17.61)	224

*Abbreviations*: *N* numbers of participants, *M* mean, *SD* standard deviation, *BPRS* the Brief Psychiatric Rating Scale, *QPR* the Questionnaire about the Process of Recovery

Educational attainment and employment status were the only two variables identified as predictors of missingness at a *p* = 0.05 level of significance, so they were included in multivariable analyses for research question 2. Pearson’s correlation revealed that the correlation between baseline variables ranged from − 0.27 to 0.39. Based on the cut-off proposed by existing literature [36], collinearity was unlikely to be a problem.

### Loneliness and objective social isolation groups

**Fig. 1:**
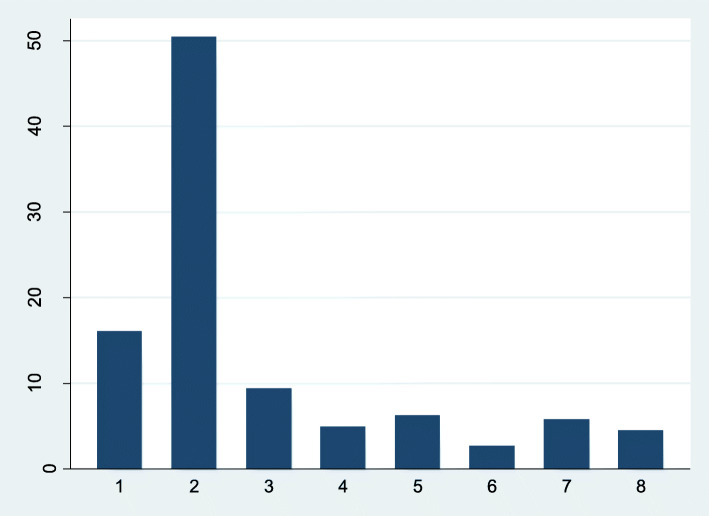
Percentage of participants in severely lonely groups. Group 1: persistently severely lonely (*N* = 36). Group 2: never severely lonely (*N* = 113). Group 3: severely lonely – not severely lonely – not severely lonely (*N* = 21). Group 4: severely lonely – severely lonely – not severely lonely (*N* = 11). Group 5: not severely lonely- severely lonely – not severely lonely (*N* = 14). Group 6: not severely lonely – severely lonely – severely lonely (*N* = 6). Group 7: not severely lonely – not severely lonely – severely lonely (*N* = 13). Group 8: severely lonely – not severely lonely – severely lonely (N = 10). Abbreviation: N = numbers of participants

The proportion of participants in each loneliness group is shown in Fig. 1. Of the 224 participants, 36 (16%) participants were in the persistently severely lonely group, 113 (50%) participants were never severely lonely, and the rest of the sample (*n* = 75, 34%) was intermittently severely lonely. Of these 75 participants in the intermittently severely lonely group, 32 participants were lonely at an earlier timepoint but not subsequently (Groups 3 and 4); 19 were not lonely at an earlier timepoint but reported being so later (Groups 6 and 7), and another 24 participants experienced fluctuating loneliness (Groups 5 and 8).

**Fig. 2:**
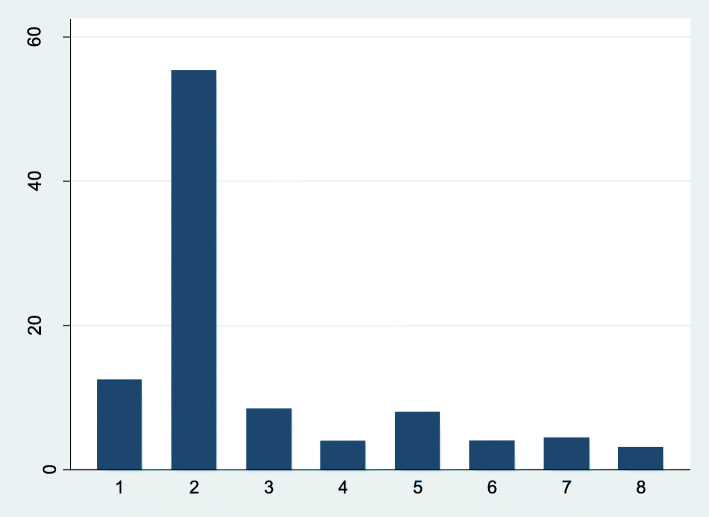
Percentage of participants in objectively socially isolated groups. Group 1: persistently objectively socially isolated (*N* = 28). Group 2: never objectively socially isolated (*N* = 124). Group 3: objectively socially isolated – not objectively socially isolated – not objectively socially isolated (*N* = 19). Group 4: objectively socially isolated – objectively socially isolated – not objectively socially isolated (N = 9). Group 5: not objectively socially isolated – objectively socially isolated – not objectively socially isolated (*N* = 18). Group 6: not objectively socially isolated – objectively socially isolated – objectively socially isolated (*N* = 9). Group 7: not objectively socially isolated – not objectively socially isolated – objectively socially isolated (*N* = 10). Group 8: objectively socially isolated – not objectively socially isolated – objectively socially isolated (N = 7). Abbreviation: N = numbers of participants

Of the same 224 participants, 28 participants (13%) met our criteria for persistent objective social isolation, 124 (55%) participants were not objectively socially isolated at any timepoint, and the rest of the sample (*n* = 72, 32%) met the criteria for the intermittent social isolation group. Of these 72 participants, a pathway out of objective social isolation was observed for 28 participants (Groups 3 and 4); a pathway into objective social isolation was observed for 19 participants (Groups 6 and 7), and another 25 participants experienced fluctuating objective social isolation (Groups 5 and 6) (Fig. 2).

### The characteristics of people in different loneliness and objective social isolation groups at baseline

The characteristics of participants in different loneliness groups are shown in Table 2. Not only were the participants in the persistent severe loneliness group more likely to be single, separated, divorced or widowed (*p* < 0.001), they were also less likely to be employed, or in education, or any full-time caring role (*p* = 0.002) than those who were never severely lonely. The persistent severe loneliness group had the smallest social network, followed by the intermittent severe loneliness group. There were significant differences in the QPR score at baseline between the three loneliness groups: persistent severe loneliness group scored the lowest on the QPR. This group was followed by the intermittent severe loneliness group. The group who was never severely lonely scored the highest on the QPR.

**Table 2:** The comparisons of baseline variables between three loneliness groups^a^

Variables	Loneliness groups							
	Persistent loneliness group (Group 1)		Intermittent loneliness group (Group 2)		Never loneliness group (Group 3)			
	Mean (SD) or %	N	Mean (SD) or %	N	Mean (SD) or %	N	***P*** value	95% CI
**Age**	40.90 (11.49)	36	36.54 (12.25)	75	41.95 (12.68)	113	Group 1 vs. Group 3: 0.66 Group 2 vs. Group 3: **0.004**^b^ Group 1 vs. Group 2: 0.08	Group 1 (37.01, 44.79) Group 2 (33.72, 39.36) Group 3 (39.58, 44.31)
**Gender**							0.25	
Male	27.78%	10	44.00%	33	40.71%	46		
Female	72.22%	26	5.00%	42	59.29%	67		
**Ethnicity**							0.77	
White British/Irish/other	62.86%	22	56.00%	42	65.49%	74		
Black, Black British/Caribbean/African/other	17.14%	6	25.33%	19	20.35%	23		
Asian, Asian British/Indian/ Pakistani/Bangladeshi/ other, Chinese	11.43%	4	9.33%	7	5.31%	6		
Mixed White/Black Caribbean, mixed White/Black African, mixed White/Asian, other mixed, other ethnic groups	8.57%	3	9.33%	7	8.85%	10		
**Marital status**							**< 0.001**	
Single/Separated/divorced/widowed	88.89%	32	86.87%	65	61.95%	70		
Married/Cohabiting	11.11%	4	13.33%	10	38.05%	43		
**UK born**							0.99	
No	25.71%	9	24.332%	18	25.00%	28		
Yes	74.29%	26	75.68%	56	75.00%	84		
**Housing**							0.49	
Permanent/supported accommodation	100%	36	96.00%	72	96.46%	109		
Unstable accommodation	0%	0	4.00%	3	3.54%	4		
**Contact with children under 16**							0.78	
No contact	2.78%	1	8.00%	6	6.19%	7		
Contact with dependent children	27.78%	10	20.00%	15	22.12%	25		
Having no children	69.44%	25	72.00%	54	71.68%	81		
**Educational attainment**							0.11	
No qualification	2.71%	9	13.33%	10	13.27%	15		
Other qualification	37.14%	13	60.00%	45	48.67%	55		
Degree	37.14%	13	26.67%	20	38.05%	43		
**Employment/education status**							**0.002**	
Not in employment/education/full time caring role	68.57%	24	46.58%	34	34.51%	39		
Yes	31.43%	11	53.42%	39	65.49%	74		
**Baseline loneliness score**	27.77 (2.13)	36	23.52 (3.76)	75	19.28 (3.15)	113	Group 1 vs. Group 3: **<.001** Group 2 vs. Group 3: **<.001** Group 1 vs. Group 2: **<.001**	Group 1 (27.05, 28.49) Group 2 (22.66, 24.39) Group 3 (18.69, 19.87)
**Baseline social network size**	3.19 (2.10)	36	4.73 (2.37)	75	5.69 (1.93)	113	Group 1 vs. Group 3: **<.001** Group 2 vs. Group 3: **0.003** Group 1 vs. Group 2: **0.001**	**Group 1 (2.49, 3.90)** **Group 2 (4.19, 5.28)** **Group 3 (5.33, 6.05)**
**Numbers of psychiatric inpatient hospitalisations**							0.49	
Never	69.44%	25	64.00%	48	59.29%	67		
Once	19.44%	7	13.33%	10	15.93%	18		
More than 2 times	11.11%	4	22.67%	17	24.78%	28		
**Number of years since first contact mental health services**							0.08	
Less than 3 months	5.56%	2	18.67%	14	17.70%	20		
3 months – 2 years	5.56%	2	22.67%	17	15.04%	17		
2–10 years	41.67%	15	32.00%	24	32.74%	37		
More than 10 years	47.22%	17	26.67%	20	34.51%	39		
**Current diagnosis**							0.09	
Psychosis or bipolar disorders^c^	19.44%	7	21.92%	16	39.09%	43		
Depression or anxiety disorders^d^	36.11%	13	30.14%	22	21.82%	24		
Personality disorders^e^	16.67%	6	13.70%	10	8.18%	9		
Other diagnosis	27.78%	10	34.25%	25	30.91%	34		
**Baseline BPRS total score**	51.19 (12.62)	36	44.77 (9.33)	75	39.79 (9.23)	112	Group 1 vs. Group 3: **<.001** Group 2 vs. Group 3: **0.0004** Group 1 vs. Group 2: **0.003**	Group 1 (46.93, 55.46) Group 2 (42.63, 46.92) Group 3 (38.06, 41.51)
**Baseline QPR total score**	35.06 (13.92)	36	49.59 (16.83)	75	57.43 (15.65)	113	Group 1 vs. Group 3: **<.001** Group 2 vs. Group 3: **0.0013** Group 1 vs. Group 2: **<.001**	Group 1 (30.34, 39.77) Group 2 (45.71, 53.46) Group 3 (54.52, 60.35)

*Abbreviations*: *SD* standard deviation, *CI* confidence interval, *N* number of participants, *BPRS* the Brief Psychiatric Rating Scale, *QPR* the Questionnaire about the Process of Recovery

^a^. t-test and chi-square test were conducted to examine the differences in baseline characteristics between three loneliness groups

^b^. significant *p*-values are marked in bold

^c^. Schizophrenia or schizoaffective disorder or bipolar affective disorder or other psychosis

^d^. Depression or Anxiety disorder or post-traumatic stress disorder

^e^. Borderline or emotionally unstable personality disorder or other personality disorder

The characteristics of participants in different social isolation groups are described in Table 3. The comparisons between the three objective social isolation groups indicated that the participants in the persistent objective social isolation group were less likely to be born in the UK, were less likely to be employed, in education or any full-time caring role (*p* = 0.01), and they were less likely to have been admitted as a psychiatric inpatient previously (*p* = 0.001), compared to the participants who were never objectively socially isolated during the study period. They were also more likely to have a diagnosis of depression, or anxiety disorder or post-traumatic stress disorder (*p* = 0.03), rather than having a diagnosis of psychosis, bipolar disorders, personality disorders or other mental health diagnoses. Statistically significant differences were also reported for their baseline loneliness score: the persistent objective social isolation group scored the highest on the ULS-8, followed by the intermittent objective social isolation group. The persistent objective social isolation group also scored lowest on the QPR. The group who was not socially isolated at any timepoint had the highest QPR score. The detailed results are shown in Table 3.

**Table 3:** The comparison of baseline variables between three objective social isolation groups^a^

Variables	Objective social isolation groups							
	Persistent social isolation group (Group 1)		Intermittent social isolation group (Group 2)		Never social isolation group (Group 3)			
	Mean (SD) or %	N	Mean (SD) or %	N	Mean (SD) or %	N	P value	95% CI
**Age**	43.76 (10.06)	28	40.29 (11.9)	72	38.92 (13.26)	124	Group 1 vs. Group 3: 0.07 Group 2 vs. Group 3: 0.47 Group 1 vs. Group 2: 0.18	Group 1 (39.86, 47.66) Group 2 (37.48, 43.10) Group 3 (36.57, 41.28)
**Gender**							0.23	
Male	25%	7	43.06%	31	41.13%	51		
Female	75%	21	56.94%	41	58.87%	73		
**Ethnicity**							0.75	
White British/Irish/other	50%	14	59.15%	42	66.13%	82		
Black, Black British/Caribbean/African/other	28.57%	8	22.54%	16	19.35%	24		
Asian, Asian British/Indian/ Pakistani/Bangladeshi/ other, Chinese	7.14%	2	8.45%	6	7.26%	9		
Mixed White/Black Caribbean, mixed White/Black African, mixed White/Asian, other mixed, other ethnic groups	14.29%	4	9.86%	7	7.26%	9		
**Marital status**							0.06	
Single/Separated/divorced/widowed	78.57%	22	83.33%	60	68.55%	85		
Married/ Cohabiting	21.43%	6	16.67%	12	31.45%	39		
**UK born**							**0.02**^b^	
No	42.86%	12	28.17%	20	18.85%	23		
Yes	57.14%	16	71.83%	51	81.25%	99		
**Housing**							0.59	
Permanent/supported accommodation	96.43%	27	98.61%	71	95.97%	119		
Unstable accommodation	3.57%	1	1.39%	1	4.03%	5		
**Contact with children under 16**							0.88	
No contact	7.14%	2	5.56%	4	6.45%	8		
Contact with dependent children	17.86%	5	26.39%	19	20.97%	26		
Having no children	75.00%	21	68.06%	49	72.58%	90		
**Educational attainment**							0.18	
No qualification	28.57%	8	15.49%	11	12.10%	15		
Other qualification	35.71%	10	47.89%	34	55.65%	69		
Degree	35.71%	10	36.62%	26	32.26%	40		
**Employment/education status**							**0.01**	
Not in employment/education/full time caring role	67.86%	19	47.14%	33	36.59%	45		
Yes	32.14%	9	52.86%	37	63.41%	78		
**Baseline loneliness score**	26.07 (3.78)	28	22.65 (4.31)	72	20.82 (4.19)	124	Group 1 vs. Group 3: **<.001** Group 2 vs. Group 3: **0.004** Group 1 vs. Group 2: **0.0004**	**Group 1 (24.61, 27.54)** **Group 2 (21.64, 23.66)** **Group 3 (20.08, 21.57)**
**Baseline social network size**	1.75 (0.97)	28	3.76 (1.78)	72	6.40 (1.50)	124	Group 1 vs. Group 3: **<.001** Group 2 vs. Group 3: **<.001** Group 1 vs. Group 2: **<.001**	**Group 1 (1.38, 2.12)** **Group 2 (3.35, 4.18)** **Group 3 (6.13, 6.66)**
**Numbers of psychiatric inpatient hospitalisations**							**0.001**	
Never	82.14%	23	45.83%	33	67.74%	84		
Once	14.29%	4	16.67%	12	15.32%	19		
More than 2 times	3.57%	1	37.50%	27	16.94%	21		
**Number of years since first contact mental health services**							0.08	
Less than 3 months	17.86%	5	13.89%	10	16.94%	21		
3 months – 2 years	17.86%	5	13.89%	10	16.94%	21		
2–10 years	32.14%	9	23.61%	17	40.32%	50		
More than 10 years	32.14%	9	48.61%	35	25.81%	32		
**Current diagnosis**							**0.03**	
Psychosis or bipolar disorder ^c^	14.81%	4	39.44%	28	28.10%	34		
Depression or anxiety disorders^d^	37.04%	10	25.35%	18	25.62%	31		
Personality disorders^e^	25.93%	7	9.86%	7	9.09%	11		
Other diagnosis	22.22%	6	25.35%	18	37.19%	45		
**Baseline BPRS total score**	49.21 (10.82)	28	44.68 (11.48)	71	41.19 (9.54)	124	Group 1 vs. Group 3: **0.0001** Group 2 vs. Group 3: **0.02** Group 1 vs. Group 2: 0.08	Group 1 (45.02, 53.41) Group 2 (41.96, 47.39) Group 3 (39.49, 42.88)
**Baseline QPR total score**	41.54 (15.74)	28	50.71 (17.44)	72	53.69 (17.46)	124	Group 1 vs. Group 3: **0.001** Group 2 vs. Group 3: 0.25 Group 1 vs. Group 2: **0.02**	**Group 1 (45.02, 53.41)** **Group 2 (50.58, 56.79)** **Group 3 (41.96, 47.39)**

*Abbreviations*: *SD* standard deviation, *CI* confidence interval, *N* number of participants, *BPRS* the Brief Psychiatric Rating Scale, *QPR* the Questionnaire about the Process of Recovery

^a^. t-test and chi-square test were conducted to examine the differences in baseline characteristics between three objective social isolation groups

^b^. significant p-values are marked in bold

^c^. Schizophrenia or schizoaffective disorder or bipolar affective disorder or other psychosis

^d^. Depression or Anxiety disorder or post-traumatic stress disorder

^e^. Borderline or emotionally unstable personality disorder or other personality disorder

### The association between loneliness groups and self-rated personal recovery at 18-month follow-up

Results from the multivariable linear regression analyses demonstrate a significant relationship between loneliness group and self-rated personal recovery at 18-month follow-up. As shown in model 1, with being never severely lonely as the reference category, intermittent severe loneliness was associated with a statistically significant 9.8-point lower score on the QPR (95% CI -13.59, − 6.02), persistent severe loneliness was associated with a 21.75-point lower score on the QPR (95% CI -26.58, − 16.93). These associations remained statistically significant in the rest of the models, after controlling for the three blocks of baseline variables (i.e., sociodemographic and psychiatric variables, and social network size) and baseline QPR. In the final model (i.e., model 5), persistent (coef. = − 12.8, 95% CI -18.83, − 6.83, *p* < 0.001) and intermittent severe loneliness (coef. = − 7.8, 95% CI -11.80, − 3.75, p < 0.001) were both associated with a lower QPR score at 18-month follow-up than for the group who did not report severe loneliness at any timepoint. One clinical variable ‘2-10 years since first contact with mental health services’ (i.e., compared with ‘less than 3 months since first contact with mental health services’ as a reference category) was also negatively associated with the QPR at 18-month follow-up in model 5. The detailed results are shown in Table 4.

**Table 4:** Linear regression between three loneliness groups and 18-month QPR, controlling for baseline variables^a^

Variables	Model 1		Model 2		Model 3		Model 4		Model 5	
	Coef. (95% CI)	P- value	Coef. (95% CI)	P-value	Coef. (95% CI)	p-value	Coef. (95% CI)	P-value	Coef. (95% CI)	p-value
**Loneliness group**										
Never severely lonely group	Reference		Reference		Reference		Reference		Reference	
Intermittently severely lonely group	−9.80 (− 13.59, −6.02)	**<.001**^b^	− 9.69 (− 13.57, −5.81)	**<.001**	−9.73 (− 13.88, −5.59)	**<.001**	−8.73 (− 12.85, − 4.61)	**<.001**	−7.78 (−11.80, − 3.75)	**<.001**
Persistently severely lonely group	−21.75 (− 26.58, − 16.93)	**<.001**	− 21.46 (− 26.70, − 16.22)	**<.001**	−19.83 (− 25.55, − 14.12)	**<.001**	−16.27 (− 22.16, − 10.37)	**<.001**	− 12.83 (−18.83, − 6.83)	**<.001**
**Psychosocial variable**										
Social network size			0.12 (−.69, .93)	0.77	0.05 (−.82, .91)	0.92	0.14 (−.72, 1.01)	0.75	0.05 (−.79, .89)	0.91
**Sociodemographic variables**										
Age (years)					−0.3 (−.18, .13)	0.75	−0.06 (−.22, .10)	0.47	−.12 (−.28, .038)	0.14
Gender (Reference - male)					0.03 (− 3.69, 3.75)	0.99	0.81 (−2.86, 4.48)	0.67	1.24 (− 2.33, 4.81)	0.49
Ethnicity										
White British/Irish/other					Reference		Reference		Reference	
Black, Black British/Caribbean/African/other					2.09 (−2.33, 6.51)	0.35	2.09 (− 2.29, 6.47)	0.35	0.53 (−3.80, 4.85)	0.81
Asian, Asian British/Indian/ Pakistani/Bangladeshi/ other, Chinese					1.23 (−5.70, 8.17)	0.73	−0.76 (−7.65, 6.14)	0.83	−.65 (−7.34, 6.03)	0.85
Mixed White/Black Caribbean, mixed White/Black African, mixed White/Asian, other mixed, other ethnic groups					−3.26 (−9.57, 3.06)	0.31	−2.92 (−9.25, 3.41)	0.36	−4.28 (−10.46, 1.90)	0.17
Employment/education status (Reference - not in employment/ education)					2.69 (−1.39, 6.76)	0.20	2.62 (−1.44, 6.69)	0.21	2.18 (−1.77, 6.13)	0.28
Educational attainment										
No qualification					Reference		Reference		Reference	
Other qualifications					1.52 (−3.94, 6.98)	0.58	1.03 (−4.40, 6.46)	0.71	.91 (−4.35, 6.18)	0.73
Degree					1.64 (−4.21, 7.49)	0.58	1.16 (−4.63, 6.96)	0.69	1.40 (−4.21, 7.02)	0.62
**Psychiatric variables**										
Number of psychiatric inpatient hospitalisations										
Never							Reference		Reference	
Once							1.87 (−2.97, 6.71)	0.45	2.16 (−2.53, 6.86)	0.36
2 or more							5.21 (.56, 9.86)	**0.03**	4.37 (−.17, 8.90)	0.059
Number of years since first contact with mental health services										
Less than 3 months							Reference		Reference	
3 months - 2 years							−3.60 (−9.87, 2.67)	0.26	−4.48 (−10.57, 1.61)	0.15
2–10 years							−8.06 (−13.51, −2.60)	**0.004**	−8.20 (− 13.49, − 2.91)	**0.003**
More than 10 years							−3.79 (−9.51, 1.92)	0.19	−4.84 (−10.41, .72)	0.09
Baseline BPRS total score							−.19 (−.38, .0002)	0.05	−0.13 (−.31, .06)	0.17
Baseline QPR total score									0.22 (.10, .33)	**<.001**
R^2^ adjusted	0.273		0.270		0.244		0.286		0.329	

*Abbreviation*: *CI* confidence interval, *N* number of participants, *BPRS* the Brief Psychiatric Rating Scale, *QPR* the Questionnaire about the Process of Recovery; R^2^ adjusted = adjusted- R^2^

^a^. multivariable linear regression analyses were conducted with QPR at 18-month follow-up as dependent variable and other factors as independent variables

^b^. significant p-values are marked in bold

### The association between objective social isolation groups and self-rated personal recovery at 18-month follow-up

Multivariable linear regression analyses demonstrate a significant relationship between the objective social isolation groups and self-rated personal recovery at 18-month follow-up. In model 1, persistent objective social isolation was the only group significantly associated with a lower QPR at 18-month follow-up (with being never objectively socially isolated as a reference category). This relationship remained statistically significant even after controlling for the three blocks of baseline variables and baseline QPR score (coef. = − 9.8, 95% CI -15.71, − 3.79, *p* = 0.001). In the final model (i.e., model 5), the QPR at 18-month follow-up was also negatively associated with ‘2-10 years and over 10 years since first contact mental health services’ (i.e., with ‘less than 3 months since first contact mental health services’ as a reference category). The detailed results are presented in Table 5.

**Table 5:** Linear regression between objective social isolation groups and 18-month QPR, controlling for baseline variablesª

Variables	Model 1		Model 2		Model 3		Model 4		Model 5	
	Coef. (95% CI)	P- value	Coef. (95% CI)	P-value	Coef. (95% CI)	p-value	Coef. (95% CI)	P-value	Coef. (95% CI)	p-value
**Social isolation group**										
Never socially isolated group	Reference		Reference		Reference		Reference		Reference	
Intermittently socially isolated group	−3.53 (−7.68, .62)	0.10	−1.75 (−5.80, 2.30)	0.40	−0.64 (− 4.77, 3.48)	0.76	−2.13 (−6.37, 2.12)	0.33	−2.23 (−6.32, 1.85)	0.28
Persistently socially isolated group	−16.35 (−22.18, − 10.53)	**<.001**^b^	−11.17 (− 17.19, − 5.16)	**<.001**	−10.63 (− 4.77, 3.48)	**0.001**	− 10.75 (− 16.93, − 4.57)	**0.001**	−9.75 (− 15.71, − 3.79)	**0.001**
**Psychosocial variable**										
Loneliness score					−.878 (− 1.33, −.43)	**<.001**	− 0.52 (− 1.01, −.04)	**0.04**	− 0.12 (−.63, .38)	0.63
**Sociodemographic variables**										
Age (years)					0.06 (−.10, .22)	0.45	0.02 (−.14, .18)	0.80	−.05 (−.22, .11)	0.51
Gender (Reference – male)					−.37 (−4.22, 3.48)	0.85	0.52 (−3.29, 4.34)	0.79	1.23 (−2.46, 4.92)	0.51
Ethnicity										
White British/Irish/other					Reference		Reference		Reference	
Black, Black British/Caribbean/African/other					2.51 (−2.08, 7.09)	0.28	2.55 (−1.99, 7.09)	0.27	0.73 (−3.73, .18)	0.75
Asian, Asian British/Indian/ Pakistani/Bangladeshi/ other, Chinese					0.89 (−6.33, 8.11)	0.81	−1.60 (−8.81, 5.61)	0.66	−1.92 (−8.85, 5.02)	0.59
Mixed White/Black Caribbean, mixed White/Black African, mixed White/Asian, other mixed, other ethnic groups					−1.62 (−8.22, 4.97)	0.63	−1.55 (−8.18, 5.08)	0.65	−3.47 (−9.91, 2.98)	0.29
Employment/education status (Reference - not in employment/ education)					4.09 (−.08, 8.25)	0.054	3.86 (−.31, 8.04)	0.07	3.08 (−.95, 7.11)	0.13
Educational attainment										
No qualification					Reference		Reference		Reference	
Other qualifications					1.30 (−4.37, 6.97)	0.65	0.59 (−5.04, 6.22)	0.84	.30 (−5.12, 5.71)	0.91
Degree					1.89 (−4.19, 7.98)	0.54	1.26 (−4.78, 7.29)	0.68	1.36 (−4.45, 7.16)	0.65
**Psychiatric variables**										
Number of psychiatric inpatient hospitalisations										
Never							Reference		Reference	
Once							2.06 (−3.00, 7.12)	0.42	2.31 (−2.57, 7.18)	0.35
More than 2 times							4.92 (−.13, 9.96)	0.056	4.50 (−.36, 9.35)	0.07
Number of years since first contact with mental health services										
Less than 3 months							Reference		Reference	
3 months - 2 years							−3.77 (−10.29, 2.75)	0.26	−4.86 (−11.14, 1.43)	0.13
2–10 years							−9.31 (−14.97, −3.64)	**0.001**	−9.56 (− 15.01, − 4.12)	**0.001**
More than 10 years							−4.56 (−10.49, 1.38)	0.13	−5.90 (− 11.64, −.15)	**0.04**
Baseline BPRS total score							−0.20 (−.41, .0002)	0.050	−0.17 (−.36, .03)	0.10
Baseline QPR total score									0.26 (.14, .38)	**<.001**
R^2^ adjusted	0.115		0.188		0.182		0.227		0.285	

*Abbreviations*: *CI* confidence interval, *N* number of participants, *BPRS* the Brief Psychiatric Rating Scale, *QPR* the Questionnaire about the Process of Recovery; R^2^ adjusted = adjusted- R^2^

^a^. multivariable linear regression analyses were conducted with QPR at 18-month follow-up as dependent variable and other factors as independent variables

^b^. significant p-values are marked in bold

## Discussion

### Sample characteristics

Our study included a diagnostically diverse sample, and the median baseline loneliness was equivalent to a moderate level of loneliness. Baseline loneliness score for our cohort is comparable to people with diagnoses across the entire spectrum of mental disorders [37, 38]. Our sample also reported having seen or heard from approximately five family members and friends in the previous month, which is comparable to previous studies of people with mental health problems [39, 40].

### Loneliness and objective social isolation groups

Whereas much research in people with psychosis or in mixed groups of mental health service users has been cross-sectional in nature, the three timepoints in this study allowed a novel exploration of associations between personal recovery and loneliness trajectory. While around 16% were lonely at all three study timepoints and 13% socially isolated at all time points, around twice as many met such criteria at only some timepoints. This suggests that a longitudinal perspective on loneliness and social isolation is valuable, as there is considerable fluctuation: as in other populations [20], it may be that some are transiently lonely or less in contact with others in response to important transitions, economic situations or fluctuations in health, while for others loneliness and social isolation may be much more enduring difficulties that they lack resources to address themselves.

While age, sex and ethnic group did not vary by loneliness trajectory (which may highlight the fact that loneliness is a universal experience that everyone may experience, regardless of one’s age, gender or ethnic background), in exploratory analyses the persistently lonely group was also more likely to be single, separated, divorced or widowed, and to be unemployed, not in education or any full-time caring role. Both marriage or cohabiting status (11% of persistently lonely vs. 38% of never lonely) and being employed at baseline (31% of persistently lonely vs. 66% of never lonely) had large associations with subsequently loneliness trajectory. These findings are in line with previous research, in which being in a stable and supportive relationship can bring positive influence on one’s physical and emotional wellbeing, for example, a sense of belonging, the feeling of be cared for and loved [41], all of which may subsequently reduce the risk of loneliness [42]. In terms of employment status, being unemployed has been linked to more financial hardships [43], which may restrict one’s access to a wide range of social activities, and workplaces may also be a significant source of friendship and social contact. The persistently lonely group also had more severe symptoms at baseline, and a large difference in self-rated recovery was already present. The group whose trajectory showed relatively severe loneliness at some but not all time points were intermediate between the persistently and never lonely groups in their initial symptom and recovery scores, and in baseline loneliness scores and social network size. Thus, at baseline, the group whose subsequent trajectory was defined at persistently lonely, were already disconnected from others in several respects, with poorer scores for both recovery and symptom severity. Prolonged loneliness has been argued to be especially significant in across age groups in the general population [20] and in our study, those who were lonely at multiple timepoints appear a group warranting attention in future research and intervention development.

People who were socially isolated at each time point, were likewise markedly more likely to be unemployed and not cohabiting or married, had more severe symptoms and rated their recovery as poorer at baseline. There was likewise a gradient between those who appeared socially isolated at each time point, those for whom this fluctuated, and those who were not isolated at any point.

### The association between loneliness groups, objective social isolation groups and personal recovery at 18-month follow-up

To the best of our knowledge, the current study is the first study examining the detrimental impact of persistent loneliness and objective social isolation on self-rated personal recovery among mental health service users. Our primary hypothesis, that people who were lonely at multiple timepoints would show less improvement in self-rated recovery was confirmed, with a persisting effect following adjustment for clinical and social variables and baseline recovery score (with recovery already poorer at baseline). This is compatible with a causal relationship between persistent loneliness and poor improvement in self-rated recovery, although the caveats below in the limitations must be noted. Social connection is conceptualised as an aspect of personal recovery [44, 45], making interpretation of their relationship over time more complex. However, our findings at least suggest that people who are and remain severely lonely and/or social isolated, with few connections with others, are a group worthy of a specific focus as they appear to recover poorly following a crisis. Despite a less prominent effect for objective social isolation on self-rated personal recovery than that for loneliness, we may speculate a shared effect of loneliness and objective social isolation on personal recovery. Loneliness and objective social isolation tend to co-occur. Not only there is a significant correlation between loneliness and objective social isolation [46], the two experiences also share some same contributing factors, including small social network and infrequent social connections with family and friends [47]. We may therefore expect that, in the current study, the majority of severely lonely participants were also socially isolated, which may further exacerbate their joint negative effect on personal recovery. The mechanisms through which persistent severe loneliness and persistent objective social isolation might impact one’s personal recovery remain unexplored. The interrelationships between a number of underlying factors, such as self-esteem, public and self-stigma, sociodemographic backgrounds, and social deprivation, may play a part.

### Strengths and limitations

The current study benefits from several main strengths: 1) we included a diagnostically varied sample which was recruited from standard secondary mental health services in the UK; 2) and they were at an illness stage that is of high clinical relevance; and 3) to the best of our knowledge, the current study is the first investigating the chronicity of loneliness and objective social isolation, and their impact on personal recovery among people with mental health problems. However, our results also subject to certain limitations: 1) our study only included mental health patients who were willing to participate in an RCT; 2) the current study had a large proportion of missing data on the diagnosis variable; 3) the attrition rate of this trial at 18-month follow-up was relatively high (i.e., 44%), therefore, the final sample of the current study only included 56% of those who completed baseline measures. Reasons for drop-out could include being unwell or having moved away, and those who dropped out may not be fully representative of those originally recruited. It is possible that this high attrition rate may compromise the generalisability of our findings. However, t-test and chi-square tests were used to compare the differences between the participants who completed measures at all three timepoints and those who did not. Educational attainment and employment status were found to be the two predictors of missingness and were subsequently controlled for in the final analysis; 4) despite the well-established scales included in the current study, the quality of our measures is subject to limitations. For example, despite being widely used in this population [5], the ULS-8 was not originally developed for people with mental health problems, and lacks clear thresholds for severe and moderate loneliness, so that we needed to specify these pragmatically for the study. In terms of the measure for social network size, given that the LSNS-6 measures both subjective and objective aspects of one’s social relationships, we had to make our own selection of items from the scale in order to focus only on objective social isolation, assessed by numbers of contracts. The measurement of loneliness at more than one timepoint is an advance over many previous studies: however, that loneliness and self-rated personal recovery were measured over the same time period and that they are relatively closely related concepts, we cannot make confident statements regarding the causal link between the two concepts.

### Research and clinical implications

Results of our study carry some important implications for future research and clinical practice. In the current study, 16% of our cohort were persistently severely lonely, and 13% suffered from persistent objective social isolation. Large-scale longitudinal cohort studies will be valuable in providing rigorous evidence concerning the extent and the impact of persistent severe loneliness and persistent objective social isolation among mental health service users. The significance of prolonged loneliness across people in different age groups in the general population has been acknowledged [20] and the current study suggests that people who are persistently lonely might also be a group of interest for further investigation and intervention development among people with longer-term mental health problems. People who are lonely or report being relatively isolated at times, but in whom improvements are observed may be a group who have more resources to remedy their own lack of connections: understanding this process better would also contribute to a greater understanding of how lack of connection may improve. Despite widespread acknowledgement of the social determinants of mental health, social interventions still tend to receive less attention in mental health care than pharmacological or psychological approaches.

Given the poor outcomes associated with persistently loneliness and social isolation, routine enquiry into loneliness and the extent of social networks as part of assessments seems desirable so that practitioners are at least aware of these potentially remediable factors. The Office for National Statistics [48] has recommended a quick screening tool, which involves three questions from the ULS-3 and a direct question as an efficient approach to identify and address loneliness in all public settings and health practices. Finally, there is a growing interest in the development of interventions targeting loneliness among mental health service users with various diagnoses [49, 50], our results suggest that targeting people who are lonely and/or socially isolated at multiple timepoints may be a fruitful approach. So far approaches to loneliness and social isolation among people with mental health problems [49] have tended to target a broad group identified as lonely and/or socially isolated on cross-sectional assessment. However, our finding that recovery from loneliness and/or social isolation at a single timepoint was frequent and outcomes were worst for those who do not recovery, fits with research in other populations suggesting that persistent loneliness is especially significant [20]. Thus, it may make sense to focus development and evaluation of interventions to reduce loneliness and/or social isolation in a mental health context on those who report being persistently lonely, with our results suggesting potential benefits for recovery. Currently there are not to our knowledge interventions with well-established effectiveness and cost-effectiveness among people with mental health problems [49], but interventions with a cognitive modification component for loneliness and supported socialisation for objective social isolation show some promise [49, 51]. Approaches found to be effective for loneliness or social isolation in other populations, such as digital interventions and support groups [52, 53], may also have potential to be adapted and tested for people with mental health problems. Our findings underscore the potential value of continuing intervention development and evaluation in this area, with people with persistent loneliness and/or social isolation a group for whom such intervention may have more potential value than in wider groups found to be lonely only at a single time point. However, although potential interventions with at least evidence, the implementation of promising interventions including interventions with a cognitive modification component and interventions that support socialisation for loneliness and objective social isolation [49], respectively, are warranted.

## Supplementary Information

**Additional file 1.** Sample characteristics at baseline.

## Data Availability

The datasets used and/or analysed during the current study are available from the corresponding author on reasonable request.
