# Supplementary material for: Trajectories of loneliness and objective social isolation and associations between persistent loneliness and self-reported personal recovery in a cohort of secondary mental health service users in the UK
Source: BMC Psychiatry. 2021 Aug 23;21:421. doi: 10.1186/s12888-021-03430-9 (PMC8381487; doi:10.1186/s12888-021-03430-9)
Supplement: Supplementary file 1 — Additional file 1. Sample characteristics at baseline. [file 12888_2021_3430_MOESM1_ESM.docx]

**Appendix**

Appendix 1. Sample characteristics at baseline

| **Variables** | **N (%) or mean (SD) or median (IQR)** |
| --- | --- |
| **Age** | 40.0 (29.90 – 49.98) |
| **Gender** |  |
| Male | 160 (40.20) |
| Female | 238 (59.80%) |
| **Ethnicity** |  |
| White British/Irish/other | 254 (63.82%) |
| Black, Black  British/Caribbean/African/other | 80 (20.10%) |
| Asian, Asian British/Indian/  Pakistani/Bangladeshi/ other,  Chinese | 30 (7.54%) |
| Mixed White/Black Caribbean,  Mixed White/Black African, mixed  White/Asian, other mixed, other  ethnic groups | 34 (8.54%) |
| **Marital status** |  |
| Single/separated/divorced/widowed | 308 (77.39%) |
| Married/cohabiting | 90 (22.61%) |
| **UK born** |  |
| Yes | 304 (77.35%) |
| No | 89 (22.65%) |
| **Housing** |  |
| Permanent/supported  accommodation | 384 (96.48%) |
| Unstable accommodation | 14 (3.52%) |
| **Contact with children under 16** |  |
| No contact | 25 (6.27%) |
| Contact with  dependent  children | 104 (26.07%) |
| Having no children | 270 (67.67%) |
| **Employment/education status** |  |
| Not in  employment  education or full time  caring role | 204 (51.91%) |
| Yes | 189 (48.09%) |
| **Educational attainment** |  |
| No qualification | 76 (19.10%) |
| School and/or vocational  qualifications | 213 (53.52%) |
| Degree | 109 (27.39%) |
| **Diagnosis** |  |
| Schizophrenia or  schizoaffective  disorder/bipolar affective  disorder/other psychosis | 124 (31.88%) |
| Depression/anxiety  disorder/post-traumatic  stress disorder | 100 (25.71%) |
| Borderline or emotionally  unstable personality  disorder/other personality  disorder | 48 (12.34%) |
| Other diagnosis | 117 (30.08%) |
| **Number of psychiatric inpatient hospitalisations** |  |
| None | 240 (60.30%) |
| Once | 60 (15.08%) |
| Twice or more | 98 (24.62%) |
| **Number of years since first contact with mental health services** |  |
| Less than 3 months | 67 (16.83%) |
| 3 months- 2 years | 67 (16.83%) |
| 2-10 years | 126 (31.66%) |
| More than 10 years | 138 (34.67%) |
| **BPRS score (24-168)** | 43 (35 – 51) |
| **QPR score (0-88)** | 53 (41 – 65) |
| **Loneliness score (8-32)** | 22 (19 – 25) |
| **Social network size (0-10)** | 4.90 (2.25) |

Abbreviations: N =number of participants; SD = standard deviation; IQR = interquartile range; BPRS = The Brief Psychiatric Rating Scale; QPR = The Questionnaire about the Process of Recovery.

For BPRS, QPR, loneliness and social network size, range of scores is shown between brackets.
